# Temperature-dependent cell death patterns induced by functionalized gold nanoparticle photothermal therapy in melanoma cells

**DOI:** 10.1038/s41598-018-26978-1

**Published:** 2018-06-07

**Authors:** Yujuan Zhang, Xuelin Zhan, Juan Xiong, Shanshan Peng, Wei Huang, Rakesh Joshi, Ying Cai, Yanling Liu, Rong Li, Keng Yuan, Nanjin Zhou, Weiping Min

**Affiliations:** 1 Institute of immunotherapy and College of Basic Medicine of Nanchang University, and Jiangxi Academy of Medical Sciences, Nanchang, China; 2 Jiangxi Provincial Key Laboratory of Immunotherapy, Nanchang, China; 3 Department of Preventive Medicine, School of Medicine, Shenzhen University, Shenzhen, China; 4 Department of Surgery, Pathology and Oncology, University of Western Ontario, London, Canada; 5 Department of Genetics and Complex Diseases, Harvard T.H. Chan School of Public Health, Boston, United States

## Abstract

Photothermal therapy (PTT) is a promising approach for cancer targeting therapy. However, the temperature-dependent killing of tumor cells in PTT remains unclear. In this study, we report necroptosis plays a role in the anti-tumor effects observed in gold nanorod (GNR)-mediated PTT in melanoma. We first synthesized gold nanorods with a targeting adaptor FA (GNRs-FA), which achieved high efficacy of targeted delivery to melanoma cells. We further demonstrated PTT, precipitated by GNRs-FA under the induction of near-infrared laser, was temperature-dependent. Furthermore, the photothermal killing of melanoma cells showed different patterns of cell death depending on varying temperature in PTT. In a lower temperature at 43 °C, the percentages of apoptosis, necroptosis and necrosis of tumor cells were 10.2%, 18.3%, and 17.6%, respectively, suggesting the cell killing is ineffective at lower temperatures. When the temperature increased to 49 °C, the cell death pattern switched to necrosis dominant (52.8%). Interestingly, when the PTT achieved a moderate temperature of 46 °C, necroptosis was significantly increased (35.1%). Additionally, GNRs-FA/PPT-mediated necroptosis was regulated by RIPK1 pathway. Taken together, this study is the first to demonstrate that temperature-dependent necroptosis is an important mechanism of inducing melanoma cell death in GNR-mediated PTT in addition to apoptosis and necrosis.

## Introduction

Cancer, especially solid tumors like melanomas, is the number one health risk to the global population. The traditional methods of cancer therapy include surgery, chemotherapy, and radiotherapy. In most clinical cases, stacked combinations of surgery, chemotherapy and radiotherapy are used to treat tumors and prevent tumor cell metastasis. However, surgery may only remove the macroscopic tumor tissue whereas smaller lesions or pockets of tumor cells may go undetected and escape removal. Surgery is also moot for removing tumor cells already in circulation in the blood or lymphatic system, or those already infiltrated into the surrounding non-cancerous tissue^1^. In contrast, although chemotherapy is a systemic treatment, it has low selectivity for tumor cells, minimal effects on the dormant but transformed tumor cells, and high systemic side effects^1–3^. Radiotherapy is local irradiation that cannot kill non-irradiated parts of tumor and long-term use can lead to an increase of resistance in selected sub-populations of cells due to a loss of sensitivity to radiation^2,3^. Therefore, new therapies for treatment of cancer, including solid tumors, are being developed in recent years.

Photothermal therapy (PTT) is a novel, safe, effective and noninvasive method for the treatment of tumors, which uses external laser light source, irradiating the targeted materials with high photothermal conversion efficiencies to achieve hyperthermia^4–9^. Additionally, tumor-targeting recognition moieties on PTT therapeutics allows them to bio-accumulate in tumor cells with minimal damage to normal cells^4–9^. In terms of induction of optimal PTT and functionalization, gold nanoparticles exhibits localized surface plasmon resonance (LSPR) of free electrons under irradiation of excitatory wavelength laser, which efficiently converts light energy into heat^4–9^. The surface plasmon resonance peak of gold nanorods (GNRs) can be tuned into the near- infrared (NIR) spectra^4,6^. NIR light is reported to penetrate into the subcutaneous cell layer up to a depth of 10 mm to a few centimeters^10^. Therefore, GNR is widely being used in PTT treatment of tumors^4,6^. After tumor-targeting modification, the PTT effects of GNRs can selectively increase the temperature of tumor tissue to 50 °C to 70 °C, exceeding the threshold that leads to the death of tumor cells and tumor-encapsulating blood vessels^11,12^.

However, the mechanism of GNRs-mediated PTT killing tumor cells remains unclear. Previous studies have shown that GNR-mediated PTT caused apoptosis and necrosis of tumor cells, which is associated with the induced temperature by PTT^13,14^. Recently, a new type of cell death termed necroptosis was identified as a novel cell death mechanism^15,16^. It has also been reported that necroptosis occurs in tumor cells after heat treatment *in vitro*^17,18^, and also could be induced by PTT in cervical cancer cell lines^19^. Recent studies have investigated the occurance of necroptosis in melanomas, especially those mediated via the mitochondria-dependent ROS-generation and induction of mitophagy/autophagy^20–22^. Interestingly, the cytoplasmic RIPK3 serine/threonine kinase has also been implicated in necroptic cell death of melanomas, but not the related RIPK1 pathway^23^. Furthermore, PTT therapy and the induction of necroptosis in cancer has also been investigated, including using gold nanoparticles^19,24–26^. Moreover, there is a wealth of pre-clinical studies supporting the justification of applying PTT in cancers, including melanomas^19,24–29^. However, investigations into temperature-dependence of necroptosis and effects in melanomas are lacking. Additionally, the relationship between temperature and necroptosis has not been clarified in spite of increased temperature serving as the critical element for PTT killing tumor cells^11,14^. Therefore, it is extremely essential to investigate the relationship between the raised temperature of PTT and necroptosis of tumor cells. In this study, we firstly investigate the influence of increased temperature on cell death in melanomas, including necroptosis for this phenomenon of GNR-mediated PTT.

## Materials and Methods

### Cell lines

A murine melanoma cell line B16-BL6 was obtained from the American Type Culture Collection (ATCC) and maintained in DMEM medium (Life Technologies, Carlsbad, CA) with 10% FBS at 37 °C in 5% CO_2_.

### Synthesis of GNR

HAuCl_4_ was acquired from SinoPharm (Shanghai Chemical Reagent Co., Ltd). Water-soluble gold nanorods were synthesized via seed-mediated growth routes as previously described^30^. Briefly, a seed solution was first prepared as follows: 1 ml of cetyltrimethylammoniumbromide (CTAB) (Sinopharm) solution (0.2 M) was mixed with 1 ml of HAuCl_4_ (0.5 mM) (Sinopharm). While the solution was stirred at 28 °C, 0.12 ml of ice-cold 0.01 M NaBH_4_ (Sinopharm) was added, until the resulting seed solution turned to brownish yellow color. Subsequently,the growth solution was prepared as follows: 50 ml of HAuCl_4_ (1 mM), 50 ml of CTAB (0.2 M) and 2.5 ml of AgNO_3_ (Sinopharm) (4 mM) were gently mixed at 28 °C, and 670 µl of ascorbic acid (0.079 M) was added until the color changed from dark yellow to colorless. To generate gold nanorods, 120 µl of the seed solution was added to the growth solution at 28 °C with gentle stirring. Within 30 min, the combined solution gradually changed color to brownish red. After 24 hrs, the solution was centrifuged at 12,000 rpm for 10 mins to remove the excessive CTAB and vacuum freeze-dried to a powder of GNR.

### Synthesis of PEI-MUA and FA-PEI-MUA

Mercaptoundecanoic acid (MUA; 654 mg) was added to 30 ml carbon tetrachloride (CCl_4_), followed by addition of 100 mmol 1-ethyl-3-[3-dimethylaminopropyl] carbodiimide hydrochloride (EDC) and 100 mmol N-Hydroxysuccinimide (NHS) at room temperature for 15 min. 100 mmol polyethylene imine (PEI) was added to the above solution at room temperature for 12 hrs. An equal volume of de-ionized water was added for extraction of water-soluble PEI-MUA. The same method was used to complete the activation of folic acid in aqueous solution. Briefly, the above water-soluble PEI-MUA solution was added in the same molar ratio with folic acid and reacted at room temperature for 12 hrs to obtain water-soluble FA-PEI-MUA complex.

### Synthesis of PEI-GNR and FA-GNR

GNR powder (HAuCl_4_; 10 mg) was added to 10 ml of the above water-soluble PEI-MUA or FA-PEI-MUA complex and stirred at room temperature for 12 hrs. The CTAB was successfully replaced by Au-S bond. An aqueous solution of PEI-GNR or FA-GNR was obtained by lyophilization, weighed and dissolved in non-ribozyme water. The plasma resonance effect was observed by a multifunctional microplate reader (SpectraMax M5e, MD, USA).

### Transmission electron microscopy (TEM)

One drop of GNR or FA-GNR complex was placed on a copper grid and allowed to dry for 10 min before examination with an electron microscope. The morphology of GNR and FA-GNR complexes were observed using TEM (LIBRA 120, Carl Zeiss, Germany).

### UV-Vis assay

The vacuum freeze-dried GNR, PEI-GNR, and FA-GNR were prepared into a 1 mg/ml homogeneous suspension, which was detected by a microplate reader (SpectraMax M5e, MD, USA) for surface plasmon resonance effect at wavelength ranges of 650 nm to 900 nm.

### Cellular uptake efficiencies of GNRs-FA

B16-BL6 cells were plated in 24-well microplates at a density of 1 × 10^5^ cells/ml. After 24 hrs, the medium was replaced with 200 µl 50 µg/ml GNR-PEI/siRNA (wt(GNR-PEI): wt(siRNA) = 32:1), 50 µg/ml GNR-FA/siRNA (wt(GNR-FA): wt(siRNA) = 32:1) or PBS/siRNA as control which were conjugated with the same amount Cy3-siRNA (0.66 µg). The nanocarriers were incubated with cells at 37 °C for 24 hrs. The cells were washed three times with PBS to remove unloaded nanoparticles and the images were taken with inverted and fluorescence microscopes. Cells were then trypsinized, harvested, washed three times with PBS to remove unloaded nanoparticles and resuspended in 200 ml of the medium and analyzed by flowcytometry Calibur (BD FACS Excalibur, BD Biosciences, Mountain View, CA).

### GNR photothermal effects *in vitro*

B16-BL6 cells were cultured (200 µl 4 × 10^4^/ml; log phase) in a 96-well plate. After 24 hrs, the B16-BL6 cells were treated with 50 µg/ml GNRs-FA or PBS as control for another 24 hrs. NIR laser irradiation (808 nm) was conducted at the varied conditions (Current (A) levels 0.55 A (0.956 W), 0.60 A (1.275 W) and 0.65 A (1.593 W)) was applied for 15 min. The laser beam targeting of specific wells in the 96-well format plates was controlled by a filter paper cover. To monitor the temperature, the laser beam was moved away, and the temperature was detected with an infrared radiation thermometer (VT02 Visual IR Thermometer, Fluke, USA) that was vertically placed at 5 cm above the target well for taking the thermal heat map very quickly (~2 Secs), and then the laser beam was moved back again.

### PTT causing the pattern of cell death assessed by MTT assay

B16-BL6 cells (200 µl 4 × 10^4^/ml; log phase) were cultured in a 96-well plate. After 24 hrs, the cells were treated in the cell culture medium with 50 µg/ml GNRs-FA or PBS as control for another 24 hrs. Caspase- or RIPK1- inhibitors Z-VAD or Nec-1 was added into the wells in final concentrations of 20 µM or with PBS as control for 4 hrs. The 808 nm NIR laser irradition at the above indicated conditions was applied for 15 min. After 24 hrs, the MTT reagent was added for another 4 hrs for conversion by mitochondrial reductase. The resulting purple crystals were dissolved in solubilization buffer and analyzed by spectrophotometry at 490 nm, using a reference of 650 nm in a microplate reader (SpectraMax M5e, MD, USA).

### Statistics

Data were presented as mean ± SD. Student’s t-test (2-tailed) was applied to determine differences between two means. For the comparison of multiple groups, one-way ANOVA test was used. For all statistical analyses, differences with p values < 0.05 were considered significant.

## Results

### Synthesis, modification and cellular uptake efficiencies of GNRs-FA

**Figure 1:**
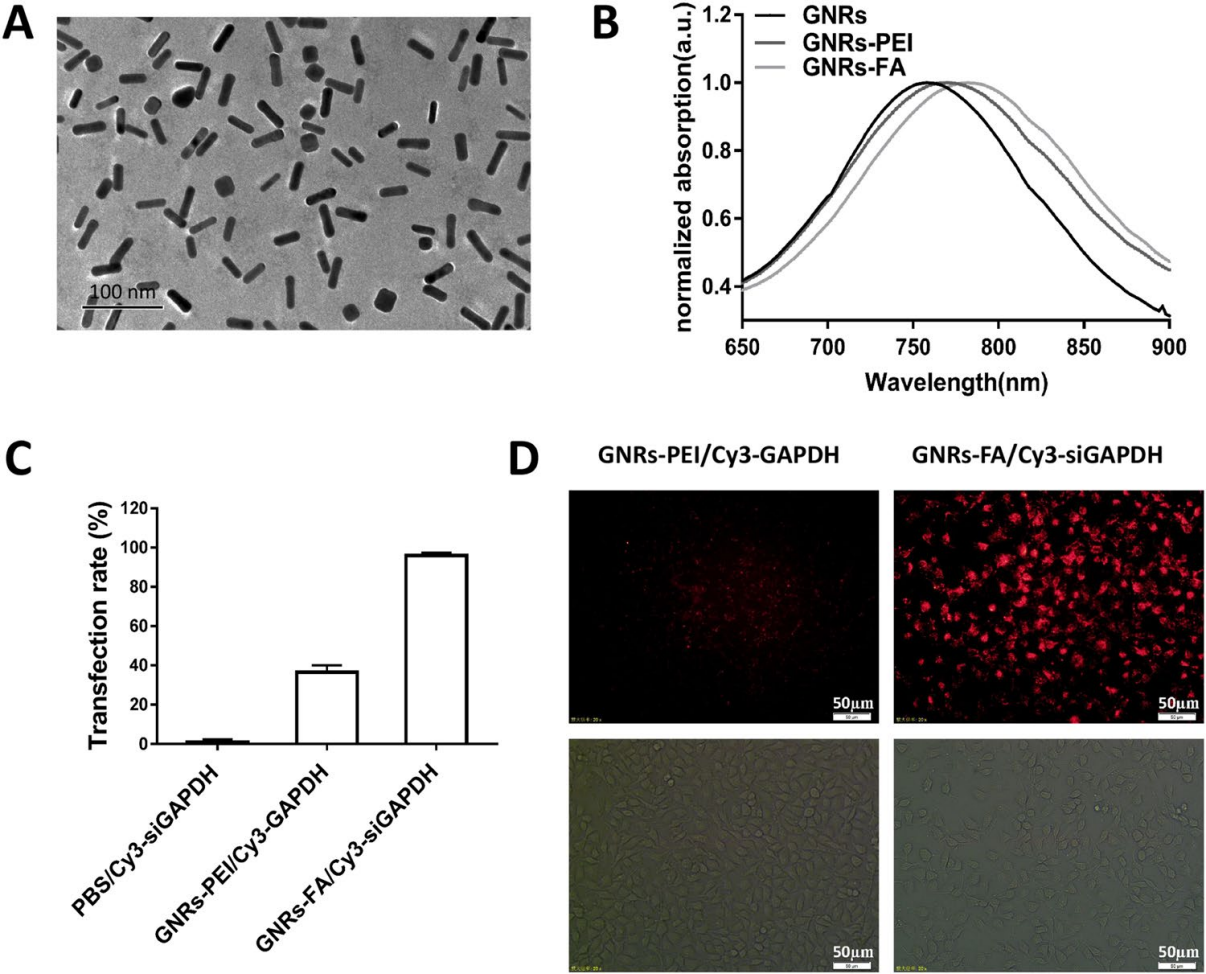
Synthesis, modification and cellular uptaking efficiencies of GNRs-FA. (**A**) TEM image of GNR after modification with folic acid (scale bar = 100 nm); (**B**) Normalized UV-Vis absorption spectra of un modified GNR or GNRs-PEI (GNRs-MUA-PEI), or GNRs-FA (GNRs-MUA-PEI-FA); (**C,D**) Transfection efficiency of Cy3-siGAPDH by PBS (control), and nanocarriers GNRs-PEI and GNRs-FA. After 24 hrs incubation, the Cy3 positive population of B16-BL6 was determined by flow cytometry (**C**), and the intracellular Cy3 fluorescence was imaged by brightfield and fluorescence microscope (**D**) (scale bar = 50 µm). Error bars represent the standard deviation of 3 experiments.

In order to make GNR-FA, we first synthesized GNR and then modified it with MUA-PEI (GNR-PEI) and conjugated with a tumor-targeting adaptor folic acid (FA in GNR-FA). The morphologies of GNRs-PEI and GNRs-FA were detected by TEM image (Fig. 1A). The dimensions of the GNRs were 10 ± 2.3 nm in radius and 40 ± 3.6 nm in length, with an average aspect ratio of 4.0. After modifications, normalized UV-Vis absorption demonstrated a spectra shift between unmodified GNR and modified GNRs-PEI (GNR-MUA-PEI) or GNRs-FA (GNR-MUA-PEI-FA) (Fig. 1B).

To test the targeted transfection efficiency of the nanoconstructs, we transfected the melanoma cell line B16-BL6 using modified GNRs that were pre-conjugated with fluorescent dye Cy3-labeled siRNA. After 24 hrs incubation, the Cy3-positive population of cells was determined by flow cytometry. Whereas non-FA targeted GNR-PEI only showed about 36.5% transfection efficacy, FA targeted transfection using GNR-FA achieved transfection efficiencies as high as 96.0% (Fig. 1C). To visually validate the intracellular uptake of Cy3-labeled siRNA transfected by nanocarriers GNR-PEI and GNR-FA, we also assessed them with brightfield and fluorescence microscopy. The intracellular fluorescence intensity in the B16-BL6 cells transected by tumor-targeted GNRs-FA was remarkably increased as compared with the cells transfected by non-targeted GNRs-PEI (Fig. 1D). These data suggested that the synthesis of GNRs-FA was successful and the targeted delivery using FA modification of GNR can achieve higher efficacies of transfection into tumor cells.

### Temperature increase induced by photothermal effects of GNRs-FA

**Figure 2:**
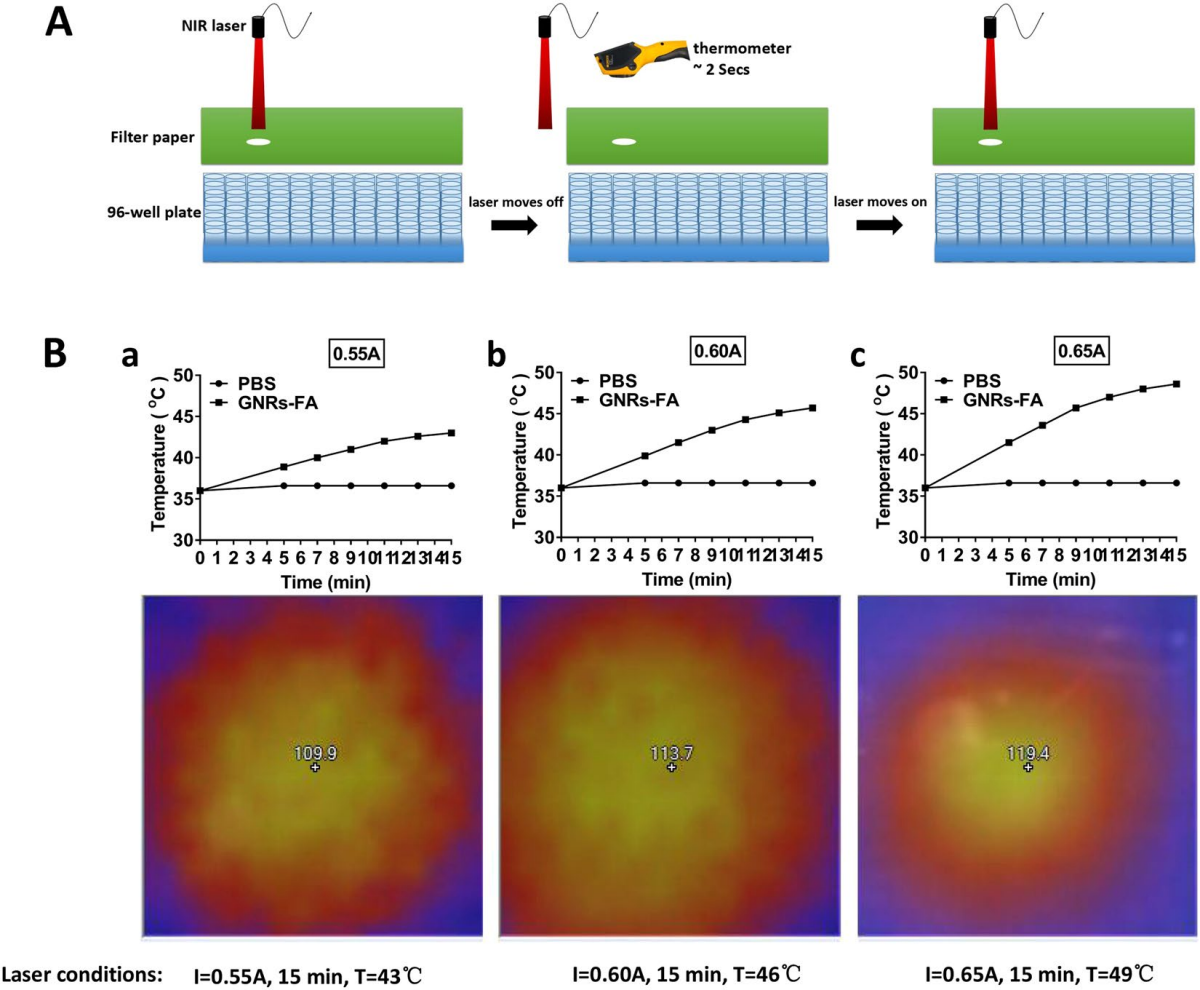
Temperature increase induced by laser-treatment of GNRs-FA. B16-BL6 cells were treated with PBS or 50 µg/ml GNRs-FA for 24 hrs. (**A**) A filter paper, with a well-sized hole for a 96-well format plate, was used to cover the treated cells to guarantee the laser irradiating the target well accurately, and an infrared radiation thermometer was applied to measure the temperature; (**B**) An 808 nm near infrared laser source was applied for 15 min with different power densities that could be adjusted by the current (0.55A (a), 0.60A (b), 0.65A (c)). The detected temperature (converted to Celsius) was plotted. Results represent 1 of 3 experiments.

Our previous study has demonstrated that GNR can produce heat under the irradiation of near infrared laser, a phenomenon called photothermal effect due to LSPR properties of the GNR. To test whether GNRs-FA can introduce efficient photothermal effects, we cultured B16-BL6 cells and transfected them with GNRs-FA, followed by the near infrared laser (808 nm) irradiation (Fig. 2A). The temperature of irradiated wells containing the cells was detected using an infrared radiation thermometer. As shown in Fig. 2B, effective photothermal effects were successfully induced by GNRs-FA under a current from 0.55A to 0.65A of laser irradiation. After 15 min with different power densities of laser power at 0.55A (Figs. 2B(a)), 0.60A (Fig. 2B(b)) and 0.65A (Fig. 2B(c)), the temperatures were increased up to 43 °C, 46 °C and 49 °C, respectively. These data implied that the increase in temperature is time- and also current-dependent, which can be controlled by adjusting the current.

### Temperature-dependent cell death patterns induced by photothermal effects of GNRs-FA in melanoma tumor cells

Photothermal treatment (PPT) produces a hyperthermal microenvironment in tumor sites, which kill tumor cells through induction of necrosis or apoptosis^13,14^. Recent studies report a new mechanism of cell death called necroptosis^15,16^. To investigate whether GNR-FA treatment is capable of inducing necroptic death in melanoma cells, we set up an experiment using a controlled amount laser beam and current intensity in order to induce temperature-dependent PTT in BL6-B16 melanoma cells (Fig. 2A). It has been reported that RIPK1-RIPK3-MLKL pathway is critical in induction of necroptosis^31^. To clarify the patterns of cell death after GNR-FA induced PTT, we treated the tumor cells with GNRs-FA and inhibitors first and then followed by laser irradiations as in Fig. 2B at three temperature levels: low (43 °C), medium (46 °C), high (49 °C).

**Figure 3:**
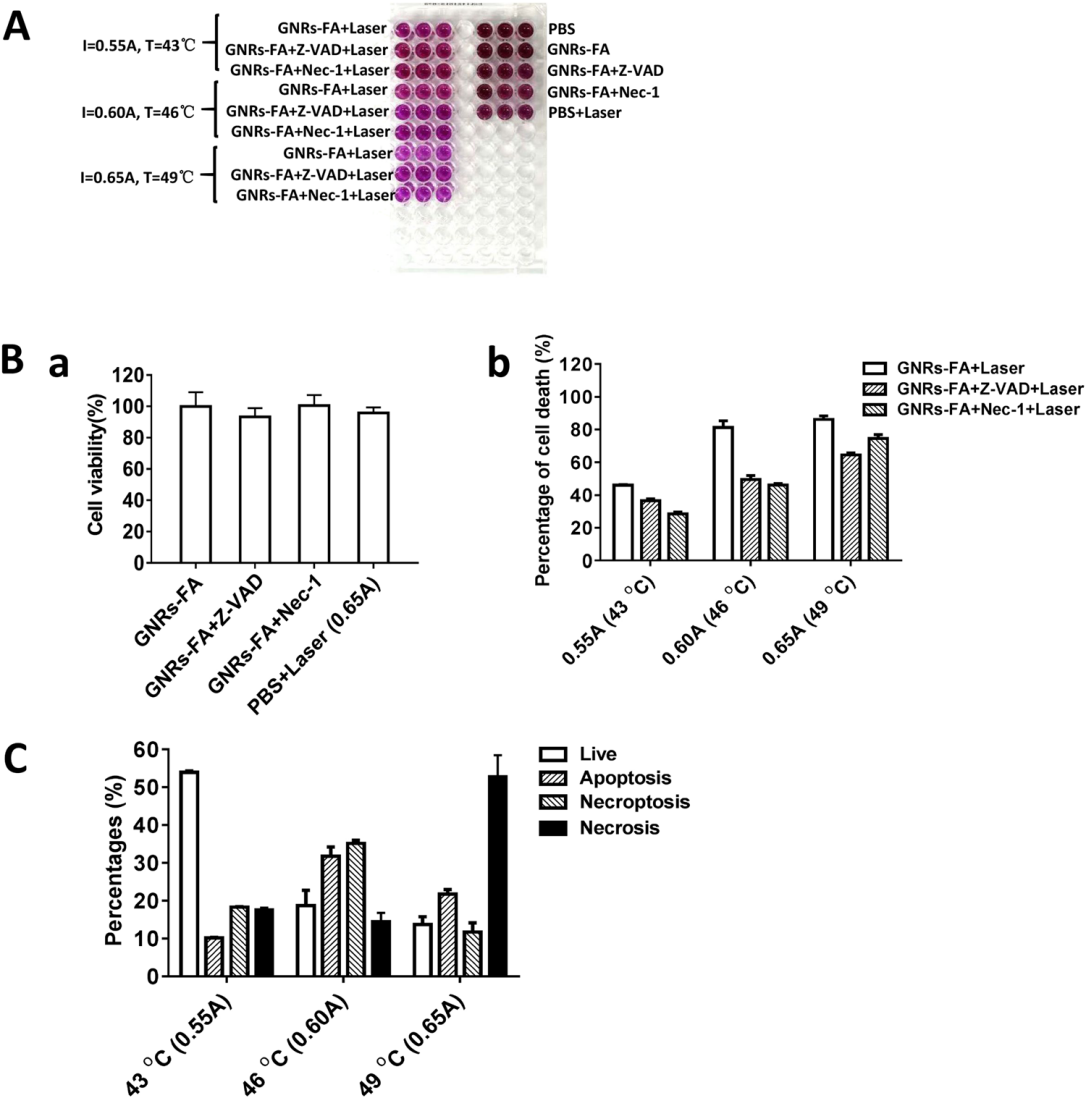
Temperature-dependent cell cytotoxicity patterns of tumor cells by GNR-mediated photothermal therapy. (**A**) B16-BL6 cells were treated GNRs-FA or PBS as control for 24 hrs. Additionally, caspase or RIPK1 inhibitor, Z-VAD or Nec-1 was implemented for 4 hrs. Finally, the cells were irradiated with laser (15 min), and cytotoxicities were evaluated by MTT assay; (**B**) (a) Cell viability (%) in each control group, and (b) cell death (%) in different treatment types were calculated; (**C**) The percentages of live, apoptotic, necroptotic, and necrotic cells at different temperature treatments (43 °C, 46 °C, 49 °C) after PTT with respective laser dosages (0.55A, 0.60A, 0.65A for 15 min). Error bars represent the standard deviation of 3 experiments (n = 3 per group/experiment).

To validate whether necroptosis and/or apoptosis are involved in the tumor killing mediated by PTT of GNRs-FA, we treated B16-BL6 using GNRs-FA or PBS, in the presence of RIPK1 inhibitor Nec-1 that blocks necroptosis pathways^31,32^. We also used pan-caspase inhibitor Z-VAD to block the apoptotic pathway^33^. After irradiation using the 808 nm NIR laser at three conditions, the cell viabilities were assessed using the MTT assay (Fig. 3A). As shown in Fig. 3B(b), the percentage of cell death decreased to 46.1% at 43 °C from 81.3% at 46 °C, and increased to 86.3% at 49 °C after the treatment with different laser doses. After, blocking necroptosis by adding Nec-1, the percentages of cell death after GNRs-FA mediated PPT at corresponding laser doses significantly dropped at low temperature (from 46.1% to 28.5%), at medium temperature (from 81.3% to 46.2%), and at high temperature (from 86.3% to 74.6%) (Fig. 3B(b)). Similar reductions were also observed when blocking the apoptosis pathway using Z-VAD, although the decreases were less at a low temperature but stronger at higher temperatures (Fig. 3B(b)).

**Figure 4:**
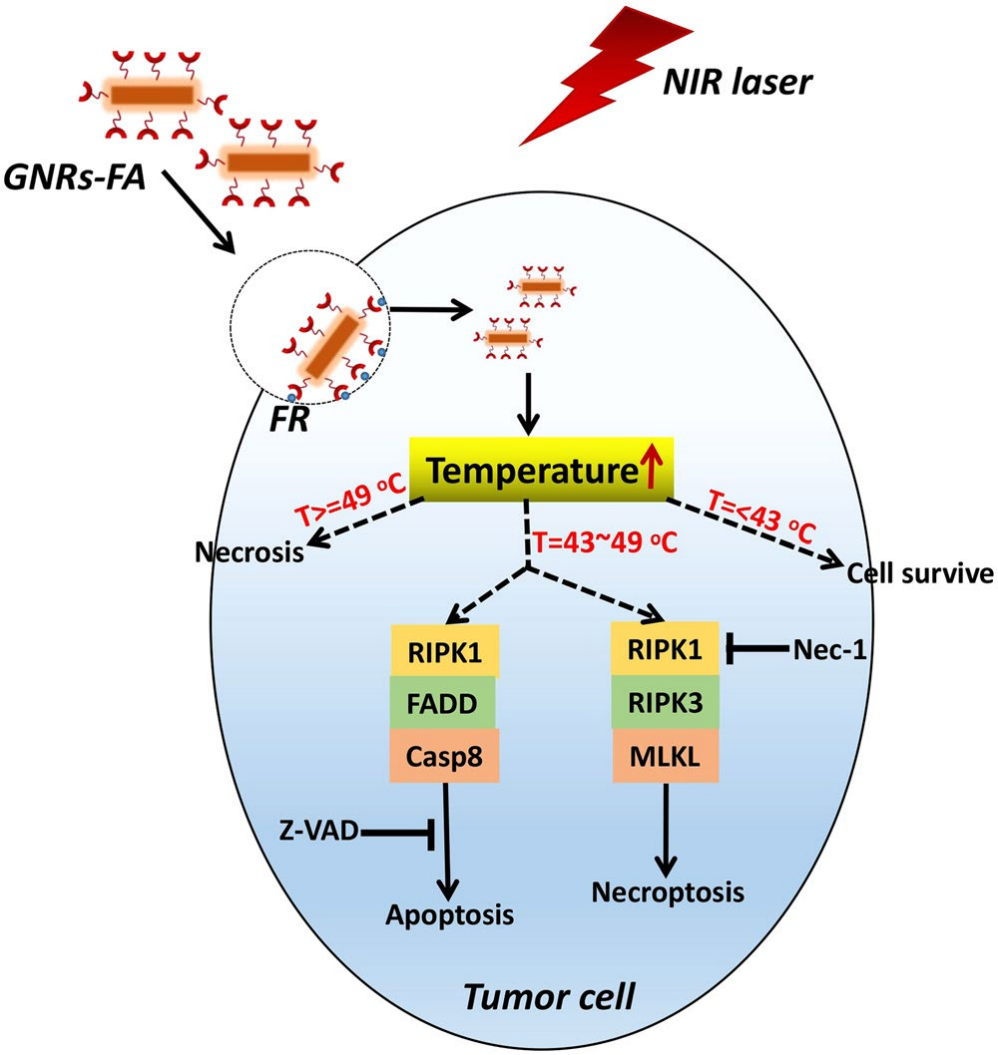
Schematic of proposed mechanism of photothermal therapy (PTT) action against tumor cells. PTT kills tumor cells mainly through necroptosis and apoptosis when the induced temperature reaches around 46 °C. Necrotic pathway is the major mechanistic pathway for PTT killing tumor cells at a higher temperature (49 °C). In contrast, most of tumor cells will survive when the induced temperature is lower than 43 °C. Error bars represent the standard deviation of 3 experiments (n = 3 per group/experiment).

Percentages of live, apoptotic, necroptotic, and necrotic cells at different temperatures (43 °C, 46 °C, 49 °C) adjusted for PTT with increasing laser doses (0.55A, 0.60A, 0.65A) for 15 min are calculated in Fig. 3(C). PTT kills tumor cells mainly through necroptosis and apoptosis when the induced temperature is 46 °C. Apoptosis, but especially necrosis, are the primary mechanisms for PTT-induced cell dath of melanoma tumor cells when the induced temperature is high (49 °C). In contrast, most tumor cells (53.9%) survived when the induced temperature is lower (43 °C (Fig. 3(C)). These data highlighted that GNR-FA induced tumor killing pattern in PPT is temperature-dependent and necroptosis regulated by RIPK1 pathway plays an important role in the killing of melanoma tumor cells by the GNRs-FA nano-construct. A proposed mechanism of photothermal therapy killing tumor cells is presented as a schematic in Fig. 4. This study provides evidence in helping us understand the mechanism of PTT killing tumor cells. RIPK1-mediated necroptosis is the other vital mechanism of photothermal therapy killing tumor cells, in addition to apoptosis and necrosis.

## Discussion

Photothermal therapy (PTT) is a novel, safe, effective and non-invasive method for the treatment of tumors when compared to the traditional cancer therapy methods of surgery and chemotherapy. Gold nanorod (GNR)-aided PTT, using near-infrared laser, enhances irradiation of tumors with higher photothermal conversion efficiency. GNR functionalized with tumor-targeting recognition moieties help it to accumulate in tumor cells, increasing the temperature-dependent cytotoxicity to the tumor cells, with minimal damage to normal cells^4–9^. Since 2005, due to the discovery of unique optical properties of gold nanoparticles (GNP) and their ability to exhibit localized surface plasmon resonance (LSPR) of free electrons under laser irradiation, GNR is widely used in PTT treatment of tumors^4–9^. El-Sayed *et al*. (2006) reported for the first time that gold nano-spheres with anti-EGFR monoclonal antibodies can target to kill malignant tumor cells by PTT *in vitro*^5^. Similarly, in 2011, Drezek *et al*. demonstrated that PTT specifically inhibited drug-resistant breast cancer cells (JIMT-1) *in vitro* by using HER2 monoclonal antibody-modified core/shell of SiO_2_/Au nanostructure^34^. We developed a new tumor-targeting FA-GNR construct and used it to evaluate anti-cancer effects on melanoma cells based on the photothermal effects for PTT^35^. In this study, we further investigated the anti-tumor mechanism on melanoma cells by PTT.

The surface plasmon resonance peak of gold nanorods (GNRs) can be tuned into the NIR band (700–1300 nm) by changing the aspect ratio of nanorod so as to reduce the emitted laser energy and increase the transmission depth^10^. After targeted modification, the PTT effects of GNRs can selectively increase the temperature of tumor tissue to 50 °C or even 70 °C, exceeding the threshold that leads to the death of tumor blood vessels or tumor cells^11,12^. PTT was demonstrated to be a promising anti-tumor approach, which also effectively killed drug-resistant cancer cells^34,36^. PTT, using gold nanoparticles, PEG-based nanoparticles and carbon nanotubes have all been used in pre-clinical studies, including for melanomas^22,25,27^. PTT using gold nanoparticles for various cancers are also in clinical trials. Gold nanoparticles with thiolated PEG and tumour necrosis factor-α (TNF-α) (CYT-6091; Aurimmune; CytImmune Sciences, Rockville, MD), have entered early clinical trials. A single-dose pilot study of AuroShell® particles (Nano- spectra Biosciences, Inc., Houston, TX) given intravenously to patients with recurrent or refractory head and neck cancer is in progress^37^. It has been reported in the past that PTT killing tumor cell is temperature-dependent, however the mechanism of cell death, necrosis, apoptosis or necroptosis, and temperature dependence has never been explored previously. Moreover, a targetable pathway, such as the RIPK pathway, in melanomas remains un-investigated. High temperatures (higher than 50 °C) primarily lead directly to necrosis, but lower ranges (below about 50 °C) primarily induce cell apoptosis^11,14,38^. For PTT applications, the range of therapy temperature is normally lower than 50 °C for causing less cell necrosis less inflammation and also protecting normal cells from high temperature surrounding^11,14,38^. So in this study, we selected three temperatures: low temperature (43 °C), medium temperature (46 °C), and high temperature (49 °C). However, the temperature dependence of new cell death mechanism necroptosis by PTT was firstly explored in this paper.

In recent years, a number of studies reported that GNR-mediated PTT can effectively inhibit the growth of tumors in mice, and the biological safety study of GNRs showed that normal mice did not display the functional damage and pathological lesions in the heart, liver, spleen, kidney, and lung after long-term therapy with GNR^38^. Our study found that percentages of survival cells were high about 53.7% at low temperature (43 °C). The lethality was apparently raised when the temperature induced by PTT was 46 °C (percentages: 18.7% live, 31.8% apoptosis, 35.1% necroptosis, 14.4% other (necrosis)), and most cells (66.9%) were killed via apoptosis and necroptosis. When the temperature was increased to 49 °C, the percentages of apoptosis and necroptosis dropped significantly, and most of cells (52.8%) were killed through necrosis, which is also indicated by previous studies^11,14,38^.

In addition, gold nanorods are also effective drug-targeting transporters. Nano drug delivery system has the advantages of improving physical and chemical properties, stability, pharmacokinetics and pharmacology and toxicology of the drug and achieve better sustained drug release, targeting and therapeutic effects^39,40^. The current research shows that gold nanorods not only can effectively load drugs but also possess the advantages of easy modification (such as targeting modifications and biological compatibility modifications) and infrared laser-induced surface plasmon resonance which can also assist drug release^41,42^. We has previously synthesized folic acid-functionalized gold nanorods capable of carrying small RNA molecules and have demonstrated that it is effective in protecting siRNAs from RNase in sera, can target folate-overexpressing tumors cells (such as melanoma, lung cancer, and breast cancer) and silence various genes (BRAF and IDO)^35^. GNR-mediated PTT combined with siRNA, miRNA and other therapeutic technologies can significantly enhance the anti-tumor effects, which opens a promising Oncotherapeutic field^35,43^.

However, the mechanism of GNR-mediated PTT killing tumor cells remains unclear. Since the 1990s, it has been discovered that the use of a pan-caspase inhibitor or knockout of caspase 8, TNF-α and FasL, no longer induces apoptosis in cells but causes necrosis^44^. In 2005, this type of necrosis,which is initiated by a specific factor and follows certain pathways and mechanisms, was named programmed necrosis or necroptosis^45^. Since 2009, the major necroptosis-activating signaling pathways have gradually been elucidated, mainly by phosphorylated RIPK1 and RIPK3 activating MLKL^31^. It was shown that the phosphorylated MLKL translocates to the cell membrane resulting in disruption of cell membrane integrity and the release of intracellular molecules that are pro-inflammatory^31^. Necroptosis accelerated the death of tumor cells and increased the sensitivity of tumor cells to anti-tumor treatment, and also killed the drug resistant tumor cells caused by dysfunction of cell apoptosis^46,47^. Parida *et al*. reported that GNRs bring about photothermal effect under NIR, leading to hyperthermic death to cervical cancer cells by necroptosis enhancing the anti-tumor effects of chemotherapeutic drug GW627368X, a selective, prostanoid EP4 inhibitor^19^. Therefore, necroptosis is an important but uncharacterized cell death mechanism, although there have been a few previous reports on tumor cell necroptosis after heat treatment *in vitro* or PTT^17–19^. This study is the first direct demonstration that temperature-dependent necroptosis is an important mechanism of inducing melanoma cell death in GNR-mediated PTT, in addition to apoptosis and necrosis. More importantly, although immunotherapeutic approaches are viable strategies for malignant melanoma therapy, the literature and our current and previous studies also demonstrated that PTT could also produce an “abscopal effect”^24,48^, which is beneficial for immunotherapy leading to a synergistic anti-tumor regimens.
